# Quantification of brain proton longitudinal relaxation (T_1_) in lithium‐treated and lithium‐naïve patients with bipolar disorder in comparison to healthy controls

**DOI:** 10.1111/bdi.12878

**Published:** 2019-12-02

**Authors:** Joe Necus, Fiona Elizabeth Smith, Peter Edward Thelwall, Carly Jay Flowers, Nishant Sinha, Peter Neal Taylor, Andrew Matthew Blamire, Yujiang Wang, David Andrew Cousins

**Affiliations:** ^1^ Translational and Clinical Research Institute Newcastle University Newcastle upon Tyne UK; ^2^ Interdisciplinary Computing and Complex BioSystems (ICOS) School of Computing Science Newcastle University Newcastle upon Tyne UK; ^3^ Newcastle Magnetic Resonance Centre Newcastle University Newcastle upon Tyne UK; ^4^ Institute of Neurology University College London London UK; ^5^ Northumberland Tyne and Wear NHS Foundation Trust Newcastle upon Tyne UK

**Keywords:** bipolar disorder, lithium, quantitative MRI, T_1_ relaxometry

## Abstract

**Background:**

Proton longitudinal relaxation (T_1_) is a quantitative MRI‐derived tissue parameter sensitive to myelin, macromolecular, iron and water content. There is some evidence to suggest that cortical T_1_ is elevated in bipolar disorder and that lithium administration reduces cortical T_1_. However, T_1_ has not yet been quantified in separate groups containing lithium‐treated patients, lithium‐naïve patients, and matched healthy controls.

**Methods:**

Euthymic patients with bipolar disorder receiving lithium (n = 18, BDL) and those on other medications but naïve to lithium (n = 20, BDC) underwent quantitative T_1_ mapping alongside healthy controls (n = 18, HC). T_1_ was compared between groups within the cortex, white matter and subcortical structures using regions of interest (ROI) derived from the Desikan‐Killiany atlas. Effect sizes for each ROI were computed for BDC vs BDL groups and Bipolar Disorder vs HC groups.

**Results:**

No significant differences in T_1_ were identified between BDL and BDC groups when corrected for multiple comparisons. Patients with bipolar disorder had significantly higher mean T_1_ in a range of ROIs compared to healthy controls, including bilateral motor, somatosensory and superior temporal regions, subcortical structures and white matter.

**Conclusions:**

The higher T_1_ values observed in the patients with bipolar disorder may reflect abnormal tissue microstructure. Whilst the precise mechanism remains unknown, these findings may have a basis in differences in myelination, macromolecular content, iron and water content between patients and controls.

## 1 INTRODUCTION

Bipolar disorder is a complex mental illness associated with multiple, potentially distinct brain structural features. Structural neuroimaging techniques have identified a number of abnormalities in bipolar disorder that have contributed towards a greater understanding of the aetiology and symptomatology of this disorder. The most common magnetic resonance imaging (MRI) techniques used to quantify structural brain changes in vivo include the use of T_1_ and T_2_ contrast‐weighted sequences, which provide high‐resolution structural information and can be used to detect volumetric changes. However, signals acquired during contrast‐weighted imaging are relative measurements, meaning that comparison of signal intensity between individuals, sites, protocols, scanners and different time points is challenging. Quantitative imaging techniques such as relaxometry^1^ enable acquisition of absolute T_1_ relaxation times. Such T_1_ measurements serve as a quantitative MRI parameter that is sensitive to myelin, macromolecular, iron and water content, more readily comparable between sites and over multiple time points.

Evidence from studies employing relaxometry suggests that patients with bipolar disorder exhibit differences in proton relaxation times within the brain. For example, longitudinal relaxation (T_1_) within the frontal and temporal lobes has been shown to be higher in patients with bipolar disorder.^2^ Higher T_1_ is also associated with lower myelin content and other microstructural changes pertinent to bipolar disorder.^3^ For instance, reductions in white matter integrity have been found to occur in bipolar disorder^4^ so the elevated T_1_ may be driven by demyelination as a feature of this disorder. Lithium, a first line treatment for bipolar disorder, is associated with greater white matter integrity^5^ but its effects on proton relaxation in bipolar disorder remains unclear. Lithium administration in healthy individuals has been shown to reduce T_1_ times in grey matter,^6^ but this may be a biophysical effect as this phenomenon is also known to occur in vitro when lithium is added to aqueous solution.^7^

Recent studies employing a related quantitative T_1_ρ mapping technique have found that T_1_ρ is higher within cerebral white matter and cerebellum in patients with bipolar disorder compared to controls.^8^ When patients were subdivided according to medication use, it was found that those prescribed lithium exhibited lower T_1_ρ values than patients taking other medications; however, this finding was not replicated in a later study.^9^

Studies have yet to quantify T_1_ across the brain of lithium‐treated and lithium‐naïve patients using high‐resolution quantitative T_1_ mapping. In this study, we use a rapid acquisition technique (DESPOT1)^10^ to quantify T_1_ within the brain of lithium‐treated and lithium‐naïve patients with bipolar disorder, together with healthy controls. We hypothesised that patients taking lithium would have lower T_1_ than those naïve to lithium and that T_1_ would be higher in patients compared to controls.

## 2 METHODS

### 2.1 Participants

Thirty‐eight euthymic subjects with a diagnosis of bipolar disorder (I or II) and 18 healthy control subjects recruited to the Bipolar Lithium Imaging and Spectroscopy Study (BLISS) were studied. Of those with bipolar disorder, 18 were taking lithium as a long‐term treatment (Bipolar Disorder Lithium, BDL) and 20 were taking other maintenance treatments but were naïve to lithium (Bipolar Disorder Control, BDC). The healthy control subjects (HC) had no history of psychiatric illness and were not taking any psychotropic medications. Subjects attended a screening visit to confirm eligibility and underwent a structured clinical interview using the NetSCID diagnostic tool (a validated online version of the Structured Clinical Interview for DSM‐5 Criteria; Telesage, Inc). Interviews and objective ratings were conducted by a trained clinical research assistant (CJF) and discussed with a senior psychiatrist (DAC). All subjects were 18 to 65 years of age and between 50 and 150 Kg in weight (upper limit determined by MRI scanner bed restrictions). Across all groups, subjects were excluded if they had a contraindication to magnetic resonance examination (including claustrophobia), a current or past medical condition deemed likely to tangibly affect brain structure, a substance use disorder (current or to a significant degree in the past; NetSCID Module E), a weekly alcohol intake exceeding 21 units (self reported), a learning disability or an impairment of capacity. Patients were excluded if they were currently liable to detention under the Mental Health Act 1983 (amended 2007). Comorbid psychiatric diagnosis in the patients, assessed using the NetSCID, was permissible (excluding neurodevelopmental, substance use as previously described and neurocognitive disorders) so long as their primary diagnosis was bipolar disorder, confirmed by a senior psychiatrist (DAC) reviewing case notes as required. Euthymic mood state was confirmed at entry to the study, defined as scores of less than seven on both the 21‐item Hamilton Depression Rating Scale (HAM‐D) and the Young Mania Rating Scale (YMRS). BDL subjects were required to have been taking lithium carbonate regularly for at least 1 year at the time of recruitment (target therapeutic range 0.6‐1.0 mmol/L) and all were taking at least one concomitant medication. BDL subjects completed the Lithium Side Effects Rating Scale (LISERS), a self‐administered scale rating the common side effects of lithium, each on a four‐point severity scale and expressed as a summated score.^11^ All scans were performed at 9 AM and the BDL subjects were instructed to take their lithium as usual the night before and submitted to a blood test immediately prior to scanning to measure their serum lithium concentration. All subjects provided written informed consent and the study was granted a favourable ethical opinion by a United Kingdom National Research Ethics Committee (14/NE/1135).

### 2.2 MRI acquisition

MR scans were performed using a 3 Tesla Philips Achieva MRI scanner (Philips Medical System) using an 8‐channel head coil. The scan protocol included:

#### 2.2.1 T_1_‐weighted imaging acquisition

3D T_1_‐weighted images (T_1_w) of brain anatomy, acquired in all subjects using the 8‐channel SENSE head coil, were obtained with a ^1^H gradient echo sequence (TR = 9.6 ms, TE = 4.6 ms, FOV = 240 × 240 × 180 mm^3^, acquisition matrix = 240 × 208 × 180, acquisition voxel size = 1 × 1.15 × 1 mm^3^, reconstructed into a matrix size of 256 × 256 × 180, 1 average).

#### 2.2.2 T_1_ parameter map acquisition

The Driven Equilibrium Single Pulse Observation of T_1_ (DESPOT1) method^10^ using two Spoiled Gradient Recalled‐Echo images (SPGR) acquired with flips angles (FA) of 4° and 15° was used to generate T_1_ parameter maps. Other image parameters were identical for each image (TR = 11.7 ms, TE = 2.4 ms, FOV = 250 × 140 × 250 mm^3^, acquisition voxel size = 0.99 × 1.0 × 2.0, reconstruction voxel size = 0.87 × 0.87 × 1.0, acquisition matrix = 252 × 250 × 140 (slices) reconstructed into 288 × 288 × 140). A B_1_ map was also acquired using a dual TR method^12^ with a conventional 3D spoiled gradient echo pulse sequence with the following parameters (nominal TR = 30ms, TR extension = 120 ms, Flip Angle = 60 degrees, FOV = 250 × 130 × 250 mm^3^).

### 2.3 Image processing and analysis

All images were exported in DICOM format and converted to NIFTI format data using the Matlab (Mathworks® Inc) toolbox ”DICOM to NIfTI”.^13^ Data pre‐processing and analysis were performed using Nipype, a Python based platform that provides a uniform interface to existing neuroimaging software and facilitates interaction between these packages within a single workflow.^14^

#### 2.3.1 T_1_w image processing

Brain tissue was sub‐divided into a series of regions of interest (ROI), or parcels, for each individual subject for analysis of regional variation in T_1_ by processing their T_1_w structural images using the FreeSurfer recon‐all pipeline (https://surfer.nmr.mgh.harvard.edu/, Version 6). Quality control of surface reconstruction was performed by visual inspection. Additional to the standard Desikan‐Killiany atlas,^15^ we also analysed results from the Destrieux^16^ atlas to demonstrate consistency in the spatial pattern of results (Supplementary Material A, Figure A1 and Table A2). Cerebellar ROI were excluded from the final analysis in order to avoid partial volume effects owing to the difficulty in accurately parcellating the cerebellar cortex. T_1_ within white matter was also compared in Montreal Neurological Institute (MNI) space using the John Hopkins University (JHU) White‐Matter Tractography ROI atlas (Supplementary Material A, Figure A3 and Table A4).^17^

#### 2.3.2 T_1_ map processing

Maps of brain proton T_1_ were generated using the DESPOT1 method.^10^ In short, by holding TR constant and acquiring two images at fixed flip angles (4° and 15°) a voxel‐wise estimation of T_1_ can be acquired based upon the relationship between signal intensity between the two images. B_1_ maps were used to correct this calculation for regional flip angle inhomogeneity. All images were visually inspected to ensure that there were no artefacts. Full details of the processing and calculation of T_1_ values are provided in Supplementary Material B.

SPGR images (FA = 4°) were linearly registered to T_1_w structural images using FMRIB's Linear Image Registration Tool to address potential movement of subjects between sequences and to derive a transformation matrix.^18^ This transformation matrix was then used to project T_1_ maps to T_1_w images in native space.

#### 2.3.3 ROI erosion

The Desikan‐Killiany atlas was used to obtain subject‐specific anatomical ROIs. Individual ROIs were eroded using the SciPy binary_erosion function (https://docs.scipy.org/, version 0.14) in order to avoid partial volume effects by removing a single outer voxel layer from each ROI. ROI effect sizes used to compare regional T_1_ between patients and controls were re‐calculated using erosion iterations ranging from zero to four in order to determine the extent to which varying degrees of ROI erosion affected the final results. The results from this, including a representative example of ROI erosion, are included in Supplementary Material C (Supplementary Figure C1). To determine tissue‐wide effects (eg, whole cortical grey matter), the corresponding ROIs were first combined and then eroded as a whole structure to then extract average tissue T1 values.

### 2.4 Statistical ROI analysis

Mean T_1_ values for each subject were calculated for voxels classified as cortical, subcortical and white matter by Freesurfer for the analysis across the different tissue types. The effects of age and sex were detrended for each group individually to quantify differences between groups (BDC, BDL and HC). This was achieved by regressing out the effects of age and sex, and adding the residuals to the mean values of the group. We chose to report detrended values rather than residuals, as the T_1_ values are in absolute units of time and should be comparable across studies. Normality of the distributions was tested and confirmed using the Lillefors test. One‐way analysis of variance (ANOVA) was used to test for group differences in detrended mean T_1_ for each tissue type, followed by post‐hoc t‐tests (applying Tukey's correction for multiple comparisons) to determine the direction of effect. The statistical significance threshold was set at *P* < .05.

For a ROI based analysis of T_1_ in region‐wise differences in mean T_1_ were determined for each Desikan‐Killiany ROI by computing effect sizes (Cohen's d) between the BDL and BDC groups. Effect sizes were computed using detrended values after regressing out the effects of age and sex for each individual ROI similar to above. Individual ROI T_1_ distributions were tested for normality using the Lillefors test, which revealed that a number of regions exhibited non‐normal distributions. Consequently, Wilcoxon signed‐rank tests were performed for each ROI, correcting for multiple comparisons across ROIs using the Benjamini‐Hochberg method.^19^

## 3 RESULTS

### 3.1 Group characteristics

Eighteen BDL subjects (10 women; mean age: 50 ± 12 SD years), 20 BDC subjects (13 women; mean age: 44 ± 12 SD years) and 18 HC subjects (11 women; mean age: 49 ± 11 SD years) were included in the analysis. The groups did not differ in mean age (*P* = .19) or sex distribution (*P* = .84) but there was a difference in duration of education (*P* < .01) (Table 1). Post‐hoc testing revealed that the HC group had remained in education for longer than the BDL group (*P* = .004) and the BDC group (*P* = .006), but the bipolar disorder groups themselves did not differ in years of education (*P* = .9). Regarding illness characteristics, the BDL and BDC groups differed in terms of duration of illness (*P* = .03), but not in terms of subtype (BD I vs II, *P* = .11) or presence of co‐morbid psychiatric diagnosis (*P* = 1). There were no group differences in YMRS scores (*P* = .06) but the groups differed in HAM‐D scores (*P* = .01), the HC group having lower scores than the BDL group (*P* = .008) and BDC group (*P* = .001), but the BDL and BDC groups did not differ from each other (and all were considered euthymic). Barring lithium, no significant differences were found between the bipolar disorder groups across all major medication classes. Medication usage information for each group, and their corresponding *P* values, are provided in Table 2.

**Table 1:** Subject characteristics

	Bipolar disorder lithium (n = 18)	Bipolar disorder control (n = 20)	Healthy control (n = 18)	Significance
Sex (M/F)	8/10	7/13	7/11	*Χ* ^2^_(1)_ = 0.36, *P* = 0.84^a^
Age (y)	50 (12)	44 (12)	49 (11)	*Χ* ^2^_(2)_ = 3.38, *P* = 0.19^b^
Educational level (y)	14 (3)	14 (2)	17 (3)	*Χ* ^2^_(2)_ = 10.54, *P* < 0.01^b^, ^c^, ^e^
YMRS score	2 (3)	1 (2)	0.2 (0.5)	*Χ* ^2^_(2)_ = 14.41, *P* = 0.06^b^, ^c^
HAM‐D score	6 (6)	5 (5)	1 (1)	*Χ* ^2^_(2)_ = 14.41, *P* < 0.01^b^, ^c^, ^f^
Bipolar disorder subtype (I/II)	9/9	5/15	n/a	*Χ* ^2^_(1)_ = 2.55, *P* = .11
Secondary diagnosis present	78%	80%	n/a	*P* = 1.00^d^
Duration of illness (y)	14.0 (10.6)	6.4 (5.3)	n/a	*U* = 91, *P* = 0.03^c^, ^e^
Duration of lithium treatment (y)	10 (7)	n/a	n/a	n/a
Priadel™ dose (mg)	828 (256)	n/a	n/a	n/a
Serum lithium concentration (mmol/L)	0.7 (0.2)	n/a	n/a	n/a
LISERS score	20 (15)	n/a	n/a	n/a

Note

Values reported as mean (standard deviation).

Abbreviations: HAM‐D, Hamilton Rating Scale for Depression; LISERS, Lithium Side Effects Rating Scale; YMRS, Young Mania Rating Scale.

^a^ Chi Square test.

^b^ Kruskall‐Wallis test.

^c^ Mann Whitney *U* test between groups.

^d^ Fisher's Exact Test.

^e^ Education; BDL vs HC *P* = .004; BDC vs HC *P* = .006; BDL vs BDC *P* = .6.

^f^ HAM‐D; BDL vs HC *P* = .008; BDC vs HC *P* = .001; BDL vs BDC *P* = .95.

**Table 2:** Medication use by class

Medication class	Bipolar disorder lithium (n = 18)	Bipolar disorder controls (n = 20)	Significance
Antipsychotics	13 (72%)	15 (75%)	OR = 1.2, *P* = .85
Antidepressants	11 (61%)	12 (61%)	OR = 1.0, *P* = .94
Anticonvulsants	5 (28%)	11 (55%)	OR = 3.2, *P* = .09
Anxiolytics	4 (22%)	6 (30%)	OR = 1.5, *P* = .72^a^
Hypnotic	5 (28%)	2 (10%)	OR = 0.3, *P* = .22^a^
Antihistamine	0	1 (5%)	OR = 0, *P* = 1.00^a^
Over the counter	0	1 (5%)	OR = 0, *P* = 1.00^a^
Mood stabilisers/ mania	18 (lithium, 100%)	0	n/a

Note

Values represent number of subjects and percentage of group. Chi Square tests found no differences in medication class between diagnostic groups other than lithium (OR: Odds Ratio) with ^a^Fisher's exact test reported when one or more cells contained an expected count lower than five.

### 3.2 Group comparison of T_1_ times across tissue types

A summary of T_1_ values subdivided according to tissue type and subject group are given in Table 3 below. Across subjects, mean T_1_ was higher in both patient groups relative to healthy controls in each tissue type.

**Table 3:** Detrended T_1_ by subject group

Tissue type	Mean detrended T_1_ in milliseconds		
	HC	BDL	BDC
Cortical	1473 (135)	1573 (140)	1582 (157)
Sub‐Cortical	1336 (128)	1428 (125)	1432 (138)
White‐Matter	985 (110)	1082 (94)	1088 (108)

Values reported as mean (standard deviation).

Abbreviations: BDC, bipolar disorder control; BDL, bipolar disorder lithium; HC, healthy control.

**Figure 1:**
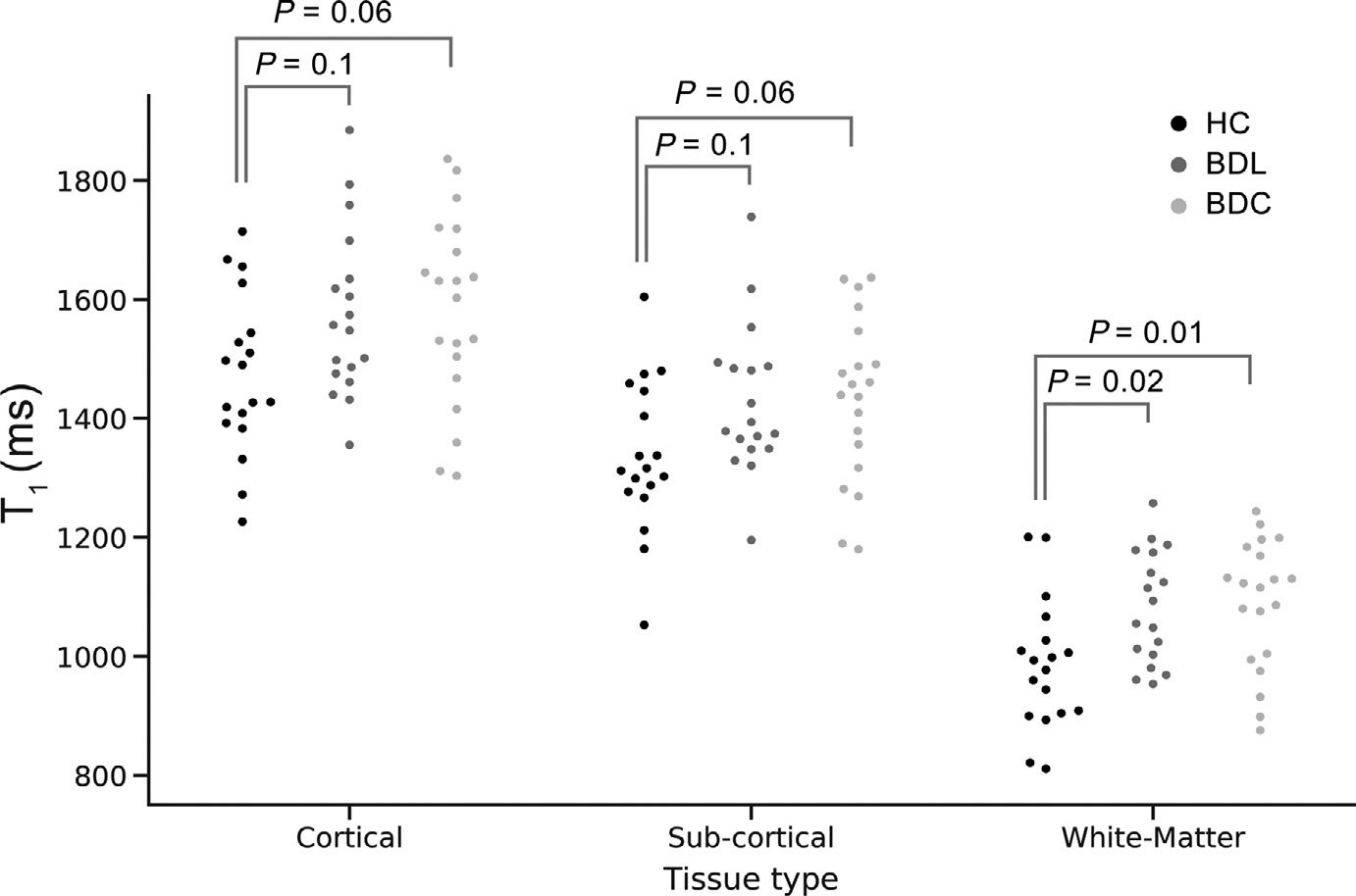
Detrended T1 values by group and tissue class. Swarm plot in which each point represents the mean detrended T_1_ for each subject in milliseconds, arranged by group and tissue class. *P*‐values represent post‐hoc (Tukey's) corrected significance

One‐way ANOVA revealed significant differences in mean T_1_ between the groups for each tissue type (Cortical F (2,52) = 3.2, *P* < .05; Subcortical F (2,52) = 3.5, *P* < .05; White matter F (2,52) = 5.7, *P* < .01). Post hoc t‐tests (applying Tukey's correction for multiple comparisons) revealed significant differences (*P* < .05) between both patient groups and healthy controls in white matter (Figure 1). Trend differences (*P* ≤ .1) were observed in mean cortical and subcortical T_1_ times. No differences in mean T_1_ values were observed between the two patient subgroups (BDL vs. BDC) in either tissue type (*P* = 1). Figure 1 shows mean detrended T_1_ per subject, subdivided by tissue type and group.

### 3.3 Investigation of regional effects

#### 3.3.1 BDL vs BDC comparison

**Figure 2:**
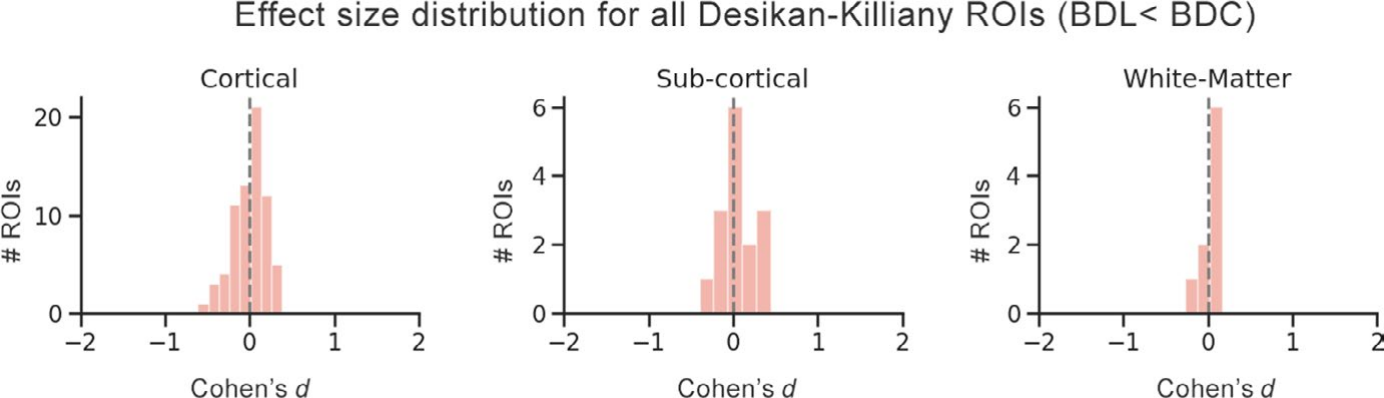
Distribution of effect sizes (BDL < BDC) for all Desikan‐Killiany regions of interest (ROI), segregated by tissue type

In order to investigate whether there were any regional differences in T_1_ times between BDL and BDC groups, effect sizes were computed for each ROI in the Desikan‐Killiany atlas. Effect sizes ranged from −0.61 to 0.45 (Figure 2), with a mean of 0.01. No ROI was significantly different between BDL and BDC groups following false discovery rate (FDR) correction.

#### 3.3.2 HC vs BD comparison using Desikan‐Killiany atlas

**Figure 3:**
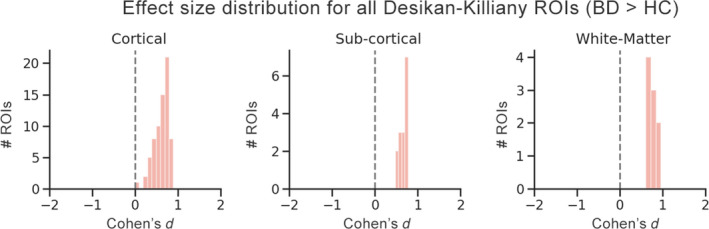
Distribution of effect sizes (BD > HC) for all Desikan‐Killiany regions of interest (ROI), segregated by tissue type

**Figure 4:**
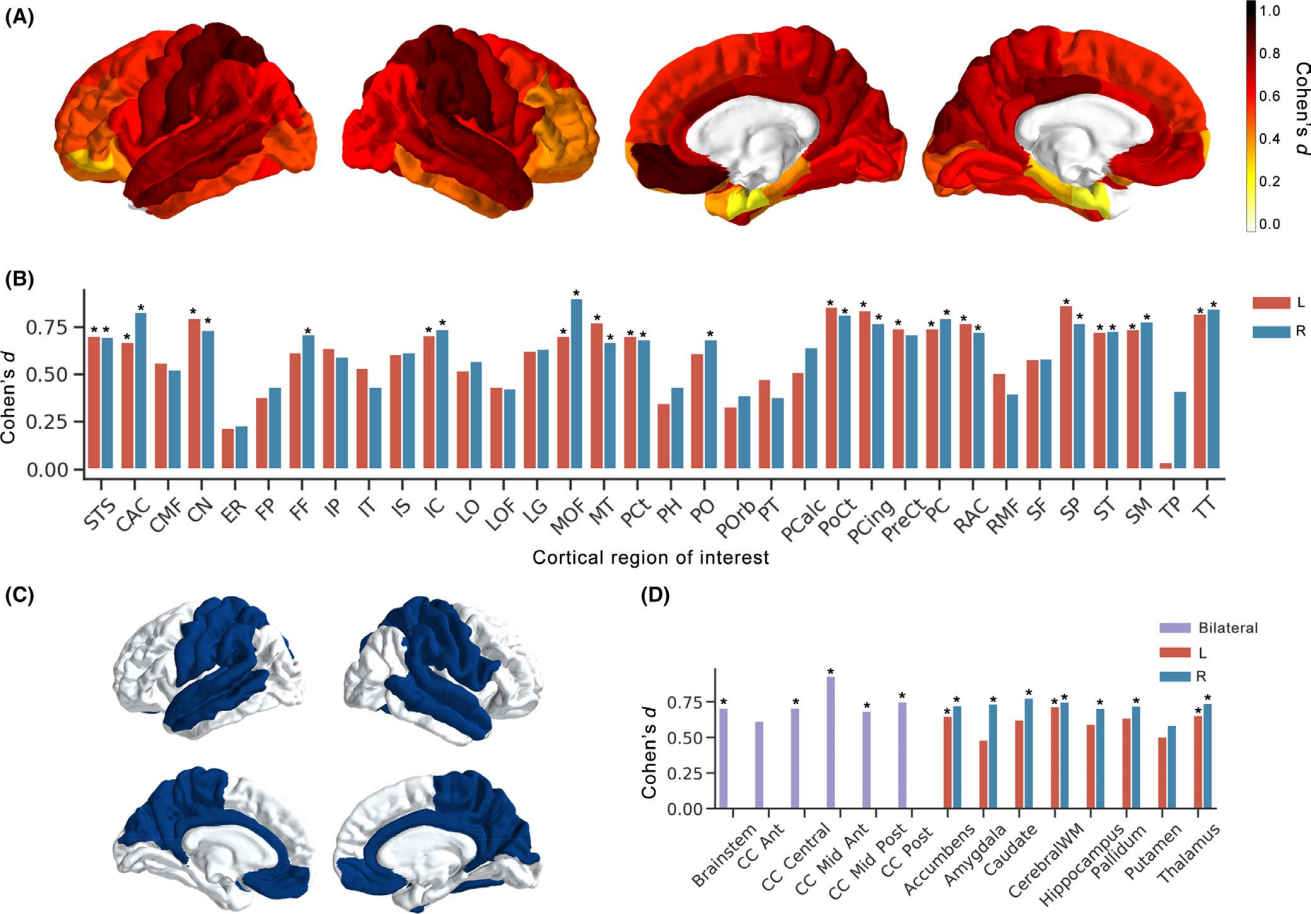
Effect sizes (Cohen's *d*) and significance testing for BD > HC comparison in Desikan‐Killiany regions of interest (ROI): A. Effect sizes per cortical ROI; B. Bar plot showing effect sizes per cortical ROI, with abbreviations are provided in Supplementary Material C; C. Cortical ROIs which remained significantly different following FDR correction for multiple comparisons (*P* < .05); D. Bar plot showing effect sizes per subcortical and WM ROI. *Indicates FDR corrected *P* < .05

We investigated the individual regional effects considering the two patient groups as a whole bipolar group (BD) in order to determine whether there are regions in which the patient group exhibits significantly higher T_1_ than healthy controls. The results from this comparison (BD > HC) revealed that all effect sizes were greater than zero (mean: 0.6 range: 0–0.97, Figure 3). Forty‐eight out of 90 ROIs survived correction for multiple comparison at a significance threshold of *P* < .05 (Figure 4). There was considerable hemispheric symmetry in terms of which regions exhibited significance, with most such cortical regions falling within the bilateral primary motor/sensory cortex and superior and middle temporal lobes (Figure 4C). A full list of effect sizes and *P* values for all ROIs is provided in Supplementary Material C (Table C1).

The effect of ROI erosion on the effect size distribution was also investigated by plotting the distribution of effect sizes across a range of ROI erosion levels (see Supplementary Material C, Supplementary Figure C2). These data suggest that partial volume effects do not significantly alter this distribution.

## 4 DISCUSSION

We report that patients with bipolar disorder have higher proton longitudinal relaxation times throughout the brain, particularly in bilateral motor, somatosensory and superior temporal regions, compared to healthy controls. Contrary to our initial hypothesis, we found no significant difference in T_1_ between patients receiving lithium and those on other medications but naïve to lithium.

This study was initially motivated by the finding that lithium administration has previously been associated with lower T_1_ in grey matter in patients and healthy controls.^2^, ^6^ It was hypothesised that patients receiving lithium treatment would exhibit shorter T_1_ compared with lithium‐naïve patients. Our results identified greater T_1_ relaxation times throughout the brain in patients with bipolar disorder relative to healthy controls, but did not find a significant difference in T_1_ between lithium‐treated and lithium‐naïve sub groups. Patients with bipolar disorder have previously been found to have elevated T_1_ values^2^ and recent work has shown that patients with first episode psychosis (but not exclusively bipolar disorder) exhibit elevated T_1_ in white matter compared with healthy controls; this effect was found to be associated with symptom severity.^20^ The elevation of T_1_ in bipolar disorder could be explained by alterations in tissue microstructure such as integrity of myelination, macromolecular content, iron and/or water content. However, due to the complexity of factors that underpin longitudinal relaxation, in vivo it is difficult to predict which factors may drive T_1_ upwards and which will cause T_1_ to decrease.

Results from our region of interest analysis show a bilaterally distributed pattern of elevated T_1_ in patients relative to controls spanning primary somatosensory, motor and superior temporal cortex. Quantitative imaging has been used to study changes associated with brain maturation during development,^21^ revealing a pattern of development beginning in primary sensory areas and moving later to the respective association cortex.^22^, ^23^ Divergence from this pattern of development has been linked to various psychiatric disorders.^24^ High resolution quantitative T_1_ mapping has also been used to characterise patterns of cortical myelination across the healthy human brain in vivo.^25^ Notably, this has revealed shorter T_1_ within primary motor, somatosensory and temporal cortices relative to the respective association cortices, indicating greater cortical myelination across these regions. This spatial pattern of cortical myelination resembles the pattern of elevated T_1_ times in our bipolar disorder subjects relative to controls. Large differences between patients and controls were seen in primary cortical regions, which likely have higher baseline levels of cortical myelination and so greater scope for change. Disruption of myelination may also prove consistent with the emerging literature demonstrating lower white matter integrity in patients with bipolar disorder.^5^, ^26^ Cortical thinning is observed in patients with bipolar disorder,^27^ and is often held to represent a reduction in grey matter volume and/or density. Apparent cortical thinning may also be explained by increased myelination, which can reduce regional T_1_ values and alter the contrast between grey and white matter, as shown in a recent combined diffusion MRI and quantitative T_1_ relaxometry study of the visual cortex in childhood.^28^ In the largest combined analysis of bipolar disorder to date, cortical thinning was most marked in frontal regions with relative sparing of primary sensorimotor areas, and whilst comparisons must be made with caution, this could arguably be the reciprocal of the distribution of elevated T_1_ values in our study. Future studies combining quantitative T_1_, dMRI and T_1_w imaging would be of great value in dissecting out the nature of cortical morphological changes in bipolar disorder.

Whilst relatively few studies have investigated brain T_1_ during bipolar disorder, a related parameter ”T_1_ρ” (T_1_‐rho)—which is sensitive to changes in T_1_ yet has different sensitivities to tissue microstructural properties—has been investigated in bipolar disorder during different mood states.^8^, ^9^ T_1_ρ was found to be elevated in euthymic patients with bipolar disorder across cerebral white matter and the cerebellum.^8^ Interestingly, that study also found reduced cerebellar T_1_ρ values in patients receiving lithium treatment. In a follow‐up study, a reduction in T_1_ρ was found in the basal ganglia during mania and depression relative to euthymia^9^ but the previous association between lithium treatment and lower T_1_ρ was not replicated. In our study we found significant elevations in T_1_ throughout the basal ganglia in euthymic bipolar patients relative to controls and it would be of interest to quantify T_1_ in non‐euthymic states in future work.

### 4.1 Strengths

To our knowledge, this is the first study to quantify regional T_1_ throughout the brain in a cohort of lithium‐treated vs lithium‐naïve patients with bipolar disorder in comparison to healthy controls. All analyses were performed on quantitative T_1_ data in subject native space, thereby avoiding non‐linear warping of the quantitative T_1_ data which tends to increase partial volume errors.^29^ In order to further minimise partial volume effects we chose to erode our subject specific regions of interest to varying degrees and compare the impact that this had upon effect size distributions. The results from this comparison indicate that our main findings were unaffected by partial volume effects. Our T_1_ values are also consistent with other quantitative studies at the same field strength,^28^ and so add to the reference ranges in health and psychiatric illness.

### 4.2 Weaknesses

Our previous report demonstrating that lithium administration reduced the T_1_ of grey matter was longitudinal^6^ whilst the current study is cross‐sectional and so vulnerable to selection bias and group differences, such as the greater duration of illness in the BDL group compared to the BDC group. Previous studies have demonstrated a relationship between duration of lithium treatment and various MRI measures, but we eschewed such an analysis as we lacked comparable data on the duration of treatment in the BDC group. A future longitudinal study in which T_1_ is quantified before and after lithium administration would be desirable.

### 4.3 Implications

We have identified a significant difference in brain proton T_1_ between bipolar patients and healthy controls. As T_1_ can readily be measured using a clinical MRI scanner, future work might explore the capacity of the technique to serve as a diagnostic biomarker, discriminating between bipolar disorder and healthy populations. Further investigation into the underlying tissue changes which give rise to elevated T_1_ may also provide insights into the pathophysiology of bipolar disorder. It is likely that treatment effects will best be explored in studies with a prospective design.

## Data Availability

The data that support the findings of this study are available from the corresponding author upon reasonable request.
