# Supplementary material for: Quantification of brain proton longitudinal relaxation (T1) in lithium‐treated and lithium‐naïve patients with bipolar disorder in comparison to healthy controls
Source: Bipolar Disord. 2019 Dec 2;23(1):41–8. doi: 10.1111/bdi.12878 (PMC7891392; doi:10.1111/bdi.12878)
Supplement: Supplementary file 1 [file BDI-23-41-s001.docx]

## Supplementary Material A

## Bipolar Disorder > Healthy Control T_1_ effect sizes using Destrieux and John Hopkins University parcellation schemes

Destrieux Atlas


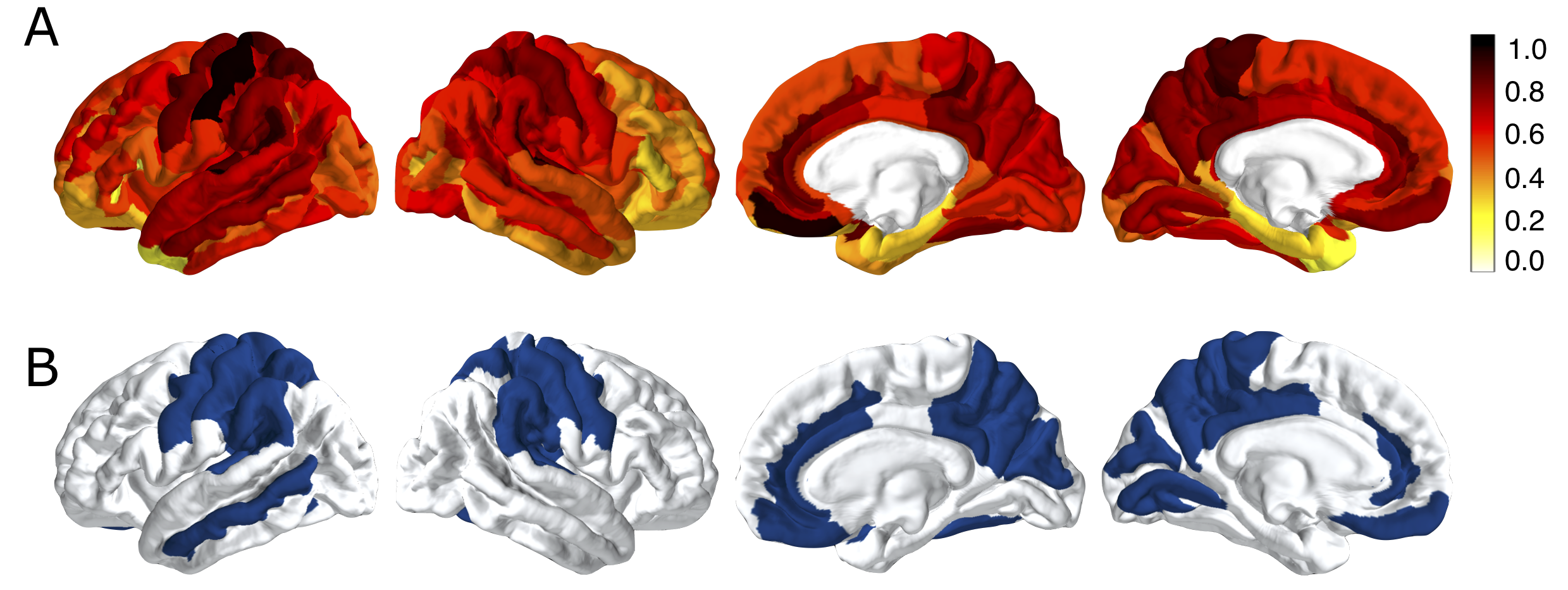


Figure A1. BD > HC T_1_ comparison using Destrieux parcellation. (A) Effect sizes per cortical ROI. (B) Cortical ROIs which remained significant following false detection rate correction for multiple comparisons (*p* < 0.05). Effect size and p-values for each ROIs are tabulated in Table S2.

Table A2: Effect size (Cohen’s d), and significance testing for BD>HC comparison for each region of interest (excluding cerebellum) in the Destrieux atlas.

| **ROI** | **Cortical** | **Left Hemisphere** | | | **Right Hemisphere** | | |
| --- | --- | --- | --- | --- | --- | --- | --- |
|  |  | **d** | **p** | **corrected_p** | **d** | **p** | **corrected_p** |
| **Accumbens-area** | Sub-cortical | 0.649 | 0.027 | 0.058 | 0.723 | 0.014 | 0.046 |
| **Amygdala** | Sub-cortical | 0.481 | 0.099 | 0.122 | 0.735 | 0.013 | 0.046 |
| **Caudate** | Sub-cortical | 0.622 | 0.034 | 0.064 | 0.776 | 0.009 | 0.046 |
| **Cerebral-White-Matter** | White-Matter | 0.717 | 0.015 | 0.046 | 0.749 | 0.012 | 0.046 |
| **G_Ins_lg_and_S_cent_ins** | Cortical | 0.667 | 0.024 | 0.053 | 0.634 | 0.031 | 0.061 |
| **G_and_S_cingul-Ant** | Cortical | 0.764 | 0.010 | 0.046 | 0.822 | 0.006 | 0.046 |
| **G_and_S_cingul-Mid-Ant** | Cortical | 0.646 | 0.028 | 0.059 | 0.741 | 0.012 | 0.046 |
| **G_and_S_cingul-Mid-Post** | Cortical | 0.695 | 0.019 | 0.048 | 0.607 | 0.039 | 0.068 |
| **G_and_S_frontomargin** | Cortical | 0.377 | 0.194 | 0.207 | 0.470 | 0.106 | 0.128 |
| **G_and_S_occipital_inf** | Cortical | 0.566 | 0.053 | 0.078 | 0.654 | 0.026 | 0.056 |
| **G_and_S_paracentral** | Cortical | 0.856 | 0.004 | 0.046 | 0.671 | 0.023 | 0.053 |
| **G_and_S_subcentral** | Cortical | 0.564 | 0.054 | 0.078 | 0.616 | 0.036 | 0.065 |
| **G_and_S_transv_frontopol** | Cortical | 0.448 | 0.123 | 0.142 | 0.585 | 0.046 | 0.073 |
| **G_cingul-Post-dorsal** | Cortical | 0.797 | 0.007 | 0.046 | 0.808 | 0.007 | 0.046 |
| **G_cingul-Post-ventral** | Cortical | 0.379 | 0.191 | 0.205 | 0.508 | 0.081 | 0.106 |
| **G_cuneus** | Cortical | 0.714 | 0.016 | 0.046 | 0.698 | 0.018 | 0.048 |
| **G_front_inf-Opercular** | Cortical | 0.491 | 0.092 | 0.116 | 0.575 | 0.050 | 0.076 |
| **G_front_inf-Orbital** | Cortical | 0.534 | 0.068 | 0.092 | 0.470 | 0.106 | 0.128 |
| **G_front_inf-Triangul** | Cortical | 0.579 | 0.048 | 0.076 | 0.319 | 0.270 | 0.277 |
| **G_front_middle** | Cortical | 0.632 | 0.032 | 0.061 | 0.390 | 0.178 | 0.194 |
| **G_front_sup** | Cortical | 0.566 | 0.053 | 0.078 | 0.539 | 0.065 | 0.092 |
| **G_insular_short** | Cortical | 0.564 | 0.054 | 0.078 | 0.511 | 0.080 | 0.105 |
| **G_oc-temp_lat-fusifor** | Cortical | 0.621 | 0.034 | 0.064 | 0.784 | 0.008 | 0.046 |
| **G_oc-temp_med-Lingual** | Cortical | 0.717 | 0.015 | 0.046 | 0.600 | 0.041 | 0.070 |
| **G_oc-temp_med-Parahip** | Cortical | 0.314 | 0.278 | 0.283 | 0.339 | 0.241 | 0.250 |
| **G_occipital_middle** | Cortical | 0.490 | 0.092 | 0.116 | 0.534 | 0.068 | 0.092 |
| **G_occipital_sup** | Cortical | 0.636 | 0.030 | 0.061 | 0.673 | 0.022 | 0.052 |
| **G_orbital** | Cortical | 0.360 | 0.214 | 0.226 | 0.354 | 0.222 | 0.231 |
| **G_pariet_inf-Angular** | Cortical | 0.628 | 0.032 | 0.062 | 0.606 | 0.039 | 0.068 |
| **G_pariet_inf-Supramar** | Cortical | 0.766 | 0.010 | 0.046 | 0.724 | 0.014 | 0.046 |
| **G_parietal_sup** | Cortical | 0.845 | 0.005 | 0.046 | 0.793 | 0.008 | 0.046 |
| **G_postcentral** | Cortical | 0.964 | 0.001 | 0.046 | 0.826 | 0.006 | 0.046 |
| **G_precentral** | Cortical | 0.818 | 0.006 | 0.046 | 0.710 | 0.016 | 0.046 |
| **G_precuneus** | Cortical | 0.780 | 0.009 | 0.046 | 0.742 | 0.012 | 0.046 |
| **G_rectus** | Cortical | 0.752 | 0.011 | 0.046 | 0.977 | 0.001 | 0.046 |
| **G_subcallosal** | Cortical | 0.662 | 0.025 | 0.054 | 0.567 | 0.053 | 0.078 |
| **G_temp_sup-G_T_transv** | Cortical | 0.871 | 0.004 | 0.046 | 0.853 | 0.004 | 0.046 |
| **G_temp_sup-Lateral** | Cortical | 0.662 | 0.025 | 0.054 | 0.517 | 0.076 | 0.103 |
| **G_temp_sup-Plan_polar** | Cortical | 0.564 | 0.054 | 0.078 | 0.866 | 0.004 | 0.046 |
| **G_temp_sup-Plan_tempo** | Cortical | 0.865 | 0.004 | 0.046 | 0.886 | 0.003 | 0.046 |
| **G_temporal_inf** | Cortical | 0.589 | 0.045 | 0.072 | 0.411 | 0.157 | 0.173 |
| **G_temporal_middle** | Cortical | 0.728 | 0.014 | 0.046 | 0.595 | 0.042 | 0.071 |
| **Hippocampus** | Sub-cortical | 0.592 | 0.043 | 0.071 | 0.705 | 0.017 | 0.046 |
| **Lat_Fis-ant-Horizont** | Cortical | 0.246 | 0.393 | 0.398 | 0.440 | 0.130 | 0.148 |
| **Lat_Fis-ant-Vertical** | Cortical | 0.183 | 0.524 | 0.524 | 0.417 | 0.153 | 0.172 |
| **Lat_Fis-post** | Cortical | 0.731 | 0.013 | 0.046 | 0.704 | 0.017 | 0.046 |
| **Pallidum** | Sub-cortical | 0.636 | 0.030 | 0.061 | 0.721 | 0.015 | 0.046 |
| **Pole_occipital** | Cortical | 0.468 | 0.107 | 0.128 | 0.598 | 0.041 | 0.070 |
| **Pole_temporal** | Cortical | 0.214 | 0.458 | 0.460 | 0.410 | 0.157 | 0.173 |
| **Putamen** | Sub-cortical | 0.502 | 0.085 | 0.109 | 0.585 | 0.046 | 0.073 |
| **S_calcarine** | Cortical | 0.516 | 0.077 | 0.103 | 0.624 | 0.034 | 0.064 |
| **S_central** | Cortical | 0.713 | 0.016 | 0.046 | 0.831 | 0.005 | 0.046 |
| **S_cingul-Marginalis** | Cortical | 0.891 | 0.003 | 0.046 | 0.867 | 0.004 | 0.046 |
| **S_circular_insula_ant** | Cortical | 0.577 | 0.049 | 0.076 | 0.381 | 0.189 | 0.204 |
| **S_circular_insula_inf** | Cortical | 0.682 | 0.021 | 0.051 | 0.818 | 0.006 | 0.046 |
| **S_circular_insula_sup** | Cortical | 0.466 | 0.109 | 0.129 | 0.592 | 0.043 | 0.071 |
| **S_collat_transv_ant** | Cortical | 0.359 | 0.215 | 0.226 | 0.703 | 0.017 | 0.046 |
| **S_collat_transv_post** | Cortical | 0.750 | 0.011 | 0.046 | 0.638 | 0.030 | 0.061 |
| **S_front_inf** | Cortical | 0.537 | 0.066 | 0.092 | 0.448 | 0.123 | 0.142 |
| **S_front_middle** | Cortical | 0.405 | 0.163 | 0.179 | 0.462 | 0.112 | 0.131 |
| **S_front_sup** | Cortical | 0.511 | 0.080 | 0.105 | 0.597 | 0.042 | 0.070 |
| **S_interm_prim-Jensen** | Cortical | 0.492 | 0.091 | 0.116 | 0.832 | 0.005 | 0.046 |
| **S_intrapariet_and_P_trans** | Cortical | 0.707 | 0.017 | 0.046 | 0.632 | 0.031 | 0.061 |
| **S_oc-temp_lat** | Cortical | 0.693 | 0.019 | 0.048 | 0.737 | 0.013 | 0.046 |
| **S_oc-temp_med_and_Lingual** | Cortical | 0.464 | 0.111 | 0.130 | 0.603 | 0.040 | 0.069 |
| **S_oc_middle_and_Lunatus** | Cortical | 0.537 | 0.066 | 0.092 | 0.355 | 0.220 | 0.231 |
| **S_oc_sup_and_transversal** | Cortical | 0.685 | 0.020 | 0.051 | 0.534 | 0.067 | 0.092 |
| **S_occipital_ant** | Cortical | 0.494 | 0.090 | 0.115 | 0.441 | 0.129 | 0.148 |
| **S_orbital-H_Shaped** | Cortical | 0.327 | 0.258 | 0.265 | 0.413 | 0.155 | 0.173 |
| **S_orbital_lateral** | Cortical | 0.424 | 0.144 | 0.163 | 0.476 | 0.102 | 0.124 |
| **S_orbital_med-olfact** | Cortical | 0.558 | 0.056 | 0.081 | 0.712 | 0.016 | 0.046 |
| **S_parieto_occipital** | Cortical | 0.484 | 0.096 | 0.120 | 0.771 | 0.009 | 0.046 |
| **S_pericallosal** | Cortical | 0.673 | 0.022 | 0.052 | 0.547 | 0.061 | 0.087 |
| **S_postcentral** | Cortical | 0.719 | 0.015 | 0.046 | 0.843 | 0.005 | 0.046 |
| **S_precentral-inf-part** | Cortical | 0.616 | 0.036 | 0.065 | 0.573 | 0.050 | 0.076 |
| **S_precentral-sup-part** | Cortical | 0.508 | 0.081 | 0.106 | 0.705 | 0.017 | 0.046 |
| **S_suborbital** | Cortical | 0.574 | 0.050 | 0.076 | 0.721 | 0.015 | 0.046 |
| **S_subparietal** | Cortical | 0.746 | 0.012 | 0.046 | 0.791 | 0.008 | 0.046 |
| **S_temporal_inf** | Cortical | 0.479 | 0.100 | 0.122 | 0.633 | 0.031 | 0.061 |
| **S_temporal_sup** | Cortical | 0.676 | 0.022 | 0.052 | 0.667 | 0.024 | 0.053 |
| **S_temporal_transverse** | Cortical | 0.968 | 0.001 | 0.046 | 1.003 | 0.001 | 0.046 |
| **Thalamus-Proper** | Sub-cortical | 0.655 | 0.026 | 0.056 | 0.739 | 0.013 | 0.046 |

John Hopkins University White Matter Atlas

T_1_ parameter maps were transformed into MNI space by applying the transformation matrix obtained from registering the structural T1w images to the standard MNI 2mm brain using FMRIB's Non Linear Image Registration Tool (Andersson et al., 2007). The ROI-wise average T1 value was extracted for each subject and compared in the following analysis.


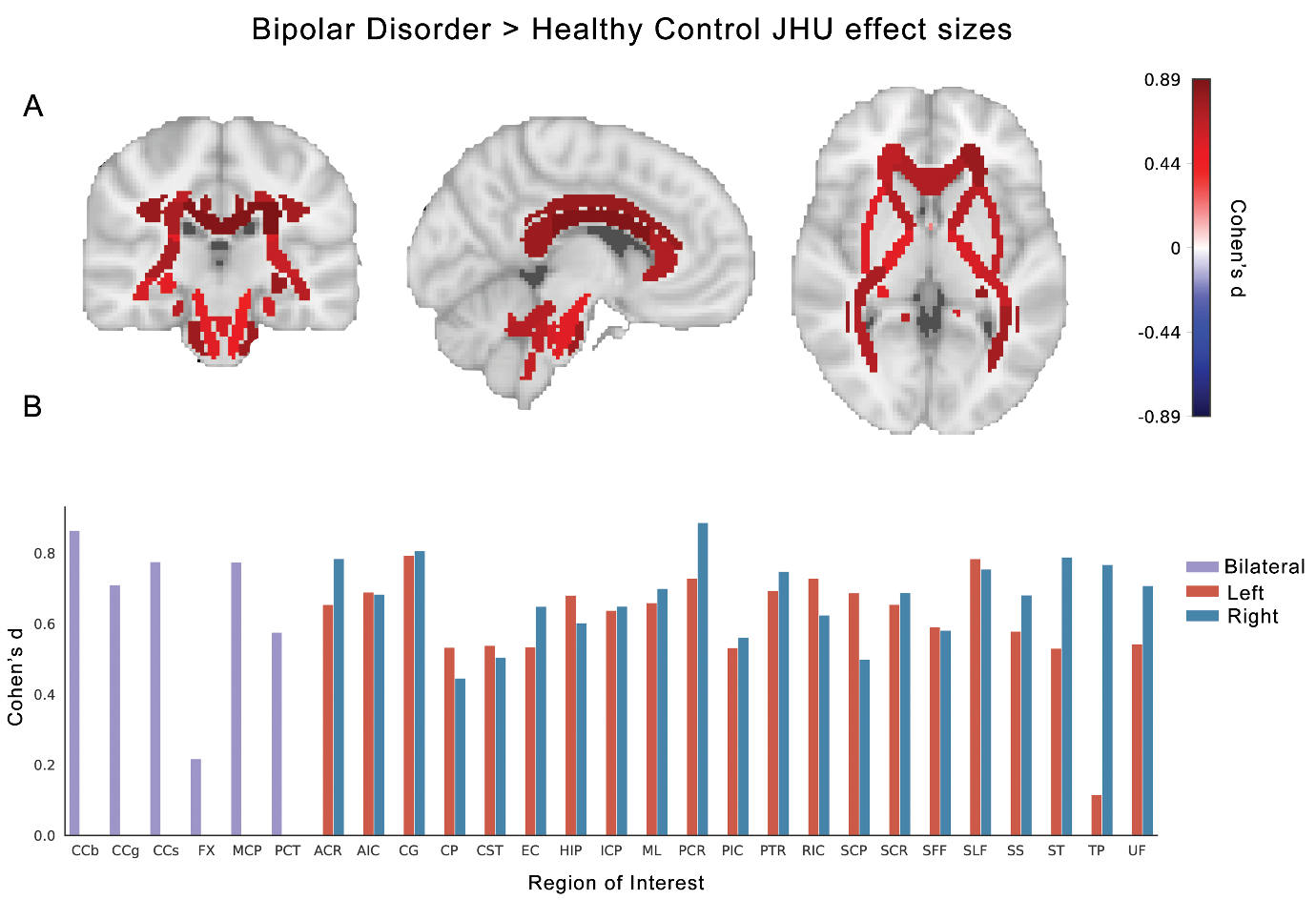


Figure A3. BD > HC T_1_ comparison using John Hopkins University (JHU) White-Matter Tractography ROI atlas. (A) Effect sizes per ROI. (B) ) Bar plot showing effect sizes per JHU ROI. Effect size and p-values for each ROIs are tabulated in Table A4.

Table A4: Effect size (Cohen’s d), and significance testing for BD>HC comparison for each region of interest in the JHU atlas.

| ROI abbreviation | ROI name | Hemisphere | Cohen's d | Original p value | FDR Corrected p value |
| --- | --- | --- | --- | --- | --- |
| ACR | Anterior corona radiata | l | 0.654 | 0.026 | 0.045 |
| ACR | Anterior corona radiata | r | 0.784 | 0.008 | 0.042 |
| AIC | Anterior limb of internal capsule | l | 0.689 | 0.019 | 0.042 |
| AIC | Anterior limb of internal capsule | r | 0.683 | 0.021 | 0.042 |
| CCb | Body of corpus callosum | bilateral | 0.864 | 0.004 | 0.042 |
| CCg | Genu of corpus callosum | bilateral | 0.710 | 0.016 | 0.042 |
| CCs | Splenium of corpus callosum | bilateral | 0.775 | 0.009 | 0.042 |
| CG | Cingulate gyrus | l | 0.793 | 0.008 | 0.042 |
| CG | Cingulate gyrus | r | 0.806 | 0.007 | 0.042 |
| CP | Cerebral peduncle | l | 0.533 | 0.068 | 0.078 |
| CP | Cerebral peduncle | r | 0.445 | 0.126 | 0.131 |
| CST | Corticospinal tract | l | 0.538 | 0.066 | 0.078 |
| CST | Corticospinal tract | r | 0.504 | 0.084 | 0.091 |
| EC | External capsule | l | 0.534 | 0.068 | 0.078 |
| EC | External capsule | r | 0.649 | 0.027 | 0.045 |
| FX | Fornix | bilateral | 0.217 | 0.451 | 0.461 |
| HIP | Hippocampus | l | 0.680 | 0.021 | 0.042 |
| HIP | Hippocampus | r | 0.602 | 0.040 | 0.060 |
| ICP | Inferior cerebellar peduncle | l | 0.637 | 0.030 | 0.048 |
| ICP | Inferior cerebellar peduncle | r | 0.649 | 0.027 | 0.045 |
| MCP | Middle Cerebellar Peduncle | bilateral | 0.774 | 0.009 | 0.042 |
| ML | Medial lemniscus | l | 0.659 | 0.025 | 0.045 |
| ML | Medial lemniscus | r | 0.699 | 0.018 | 0.042 |
| PCR | Posterior corona radiata | l | 0.728 | 0.014 | 0.042 |
| PCR | Posterior corona radiata | r | 0.886 | 0.003 | 0.042 |
| PCT | Pontine Crossing Tract | bilateral | 0.575 | 0.049 | 0.066 |
| PIC | Posterior limb of internal capsule | l | 0.531 | 0.069 | 0.078 |
| PIC | Posterior limb of internal capsule | r | 0.561 | 0.055 | 0.072 |
| PTR | Posterior thalamic radiation | l | 0.694 | 0.019 | 0.042 |
| PTR | Posterior thalamic radiation | r | 0.747 | 0.012 | 0.042 |
| RIC | Retrolenticular part of internal capsule | l | 0.728 | 0.014 | 0.042 |
| RIC | Retrolenticular part of internal capsule | r | 0.624 | 0.034 | 0.052 |
| SCP | Superior cerebellar peduncle | l | 0.687 | 0.020 | 0.042 |
| SCP | Superior cerebellar peduncle | r | 0.499 | 0.087 | 0.093 |
| SCR | Superior corona radiata | l | 0.654 | 0.026 | 0.045 |
| SCR | Superior corona radiata | r | 0.687 | 0.020 | 0.042 |
| SFF | Superior fronto-occipital fasciculus | l | 0.590 | 0.044 | 0.064 |
| SFF | Superior fronto-occipital fasciculus | r | 0.581 | 0.047 | 0.066 |
| SLF | Superior longitudinal fasciculus | l | 0.784 | 0.008 | 0.042 |
| SLF | Superior longitudinal fasciculus | r | 0.754 | 0.011 | 0.042 |
| SS | Sagittal stratum | l | 0.578 | 0.048 | 0.066 |
| SS | Sagittal stratum | r | 0.681 | 0.021 | 0.042 |
| ST | Stria terminalis | l | 0.530 | 0.070 | 0.078 |
| ST | Stria terminalis | r | 0.788 | 0.008 | 0.042 |
| TP | Tapetum | l | 0.115 | 0.688 | 0.688 |
| TP | Tapetum | r | 0.767 | 0.010 | 0.042 |
| UF | Uncinate fasciculus | l | 0.542 | 0.063 | 0.078 |
| UF | Uncinate fasciculus | r | 0.707 | 0.017 | 0.042 |
