# Supplementary material for: Quantification of brain proton longitudinal relaxation (T1) in lithium‐treated and lithium‐naïve patients with bipolar disorder in comparison to healthy controls
Source: Bipolar Disord. 2019 Dec 2;23(1):41–8. doi: 10.1111/bdi.12878 (PMC7891392; doi:10.1111/bdi.12878)
Supplement: Supplementary file 2 [file BDI-23-41-s002.docx]

## Supplementary Material B

Quantitative relaxometry refers to the quantification of relaxation times, such as T_1_ and T_2_. Unlike, T1 and T2 weighted images, which modulate acquisition sequences in order to optimise image contrast, signals acquired during quantitative MRI sequences (qMRI) directly correspond to an absolute quantitative measure of time (Deoni, 2010).

The gold standard method used to quantify T_1_ and T_2_ relaxation involves the use of an acquisition sequence known as “Inversion Recovery”. Inversion recovery sequences characterise relaxation times by inverting the magnetic moment of protons using a radiofrequency (RF) pulse and measuring the time taken for net magnetisation to fully recover. To achieve this, a 180° RF pulse is used to fully invert the net magnetisation vector (Figure 1A). Over time this net magnetisation starts to recover at a rate determined by T_1_, during which time the net magnetisation will pass through a null point (Figure 1B). In order to characterise the T_1_ recovery curve, a single 90° RF pulse is applied during each recovery at varying intervals. Through repetition of this process, the extent of recovery may be determined by recording and plotting the net magnetisation following this RF pulse. For example, if a 90° pulse is applied at the null point, net magnetisation becomes fully inverted once again, whereas if a 90° pulse is applied following full recovery (Figure 1C), net magnetisation will return to zero.

Figure 1. Following a 180° RF pulse net magnetisation is inverted (A), during recovery this passes through a null point (B) before finally recovering to equilibrium magnetisation (C). The time taken to fully recover is the T_1_ time.

Whilst inversion recovery sequences are able to accurately quantify relaxation time, this process takes a long time as it requires waiting for the full recovery of magnetisation. In order to overcome this, a range of newer acquisition protocols have been developed which enable an estimation of relaxation rates whilst minimising acquisition time.

Driven Equilibrium Single Pulse Observation of T_1_ (DESPOT1) is an example of such a sequence (Deoni et al.,2005). DESPOT1 works on the basis that signal intensity in Spoiled Gradient Recalled-Echo (SPGR) images (SI_SPGR_) can be modelled on equations relating the density of protons within a given voxel (proton density), longitudinal relaxation (T_1_), the length of time between the application of excitation pulses (repetition time, TR), the rotation of the net magnetisation vector (flip angle, α) and, M_0_, a factor proportional to the equilibrium longitudinal magnetisation (Equation 1.1).

Equation 1.1

Using the DESPOT1 approach, T_1_ parameter maps are produced via the acquisition of two consecutive images during which the TR is held constant but the flip angle is modified. This means that two values (x1, y1 and x2, y2, Equation 1.2) are obtained for which the slope between these points is equivalent to E_1_ (Equation 1.3).

Equation 1.2

Equation 1.3

As a result, by rearranging E_1_ to the form shown in Equation 1.4, T_1_ may be calculated by measuring the slope of this line.

Equation 1.4

Quantitative T_1_ images can be improved by correcting for flip angle inaccuracies resulting from B1 field inhomogeneity (Deoni, 2007). During the present study this was achieved via direct mapping of the B1 field magnitude and using this acquisition to correct flip angles in Equations 1.2.

Uses of qMRI

The development of rapid acquisition quantitative sequences means that qMRI is now becoming feasible in a clinical context. One of the major advantages of qMRI over qualitative imaging includes the ability to directly compare image signal intensity over multiple time points and between acquisition sites. T_1_ and T_2_ relaxation serve as quantitative measures which are sensitive to myelin, macromolecular content, iron and water content. As a result, changes in relaxation may act as biomarkers which represent an underlying pathology. For example, studies have used quantitative relaxometry to identify changes in iron content during Parkinson’s disease (Lee et al., 2018), visualise excess water content in malignant gliomas (Blystad et al., 2017), map demyelination during multiple sclerosis (Manfredonia et al., 2007), and detect hippocampal sclerosis during temporal lobe epilepsy (Jackson et al.,1993).

References

Blystad, I., Warntjes, J. B. M., Smedby, O., Lundberg, P., Larsson, E. M., and Tisell, A. (2017). Quantitative MRI for analysis of peritumoral edema in malignant gliomas. PLoS ONE, 12(5).

Deoni, S. C. (2007). High‐resolution T1 mapping of the brain at 3T with driven equilibrium single pulse observation of T1 with high‐speed incorporation of RF field inhomogeneities (DESPOT1‐HIFI). J. Magn. Reson. Imaging, 26: 1106-1111. doi:10.1002/jmri.21130

Deoni, S. C. L., Peters, T. M., and Rutt, B. K. (2005). High-resolution T-1 and T-2 mapping of the brain in a clinically acceptable time with DESPOT1 and DESPOT2. Magnetic Resonance in Medicine, 53(1):237–241.

Deoni, S. C. (2010). Quantitative relaxometry of the brain. Top Magn Reson Imaging, 21(2):101–113.

Jackson, G. D., Connelly, A., Duncan, J. S., Grunewald, R. A., and Gadian, D. G. (1993). Detection of hippocampal pathology in intractable partial epilepsy: increased sensitivity with quantitative magnetic resonance T2 relaxometry. Neurology, 43(9):1793–1799.

Lee, H., Baek, S. Y., Chun, S. Y., Lee, J. H., and Cho, H. (2018). Specific visualization of neuromelanin-iron complex and ferric iron in the human post-mortem substantia nigra using MR relaxometry at 7T. Neuroimage, 172:874–885.

Manfredonia, F., Ciccarelli, O., Khaleeli, Z., Tozer, D. J., Sastre-Garriga, J., Miller, D. H., and Thompson, A. J. (2007). Normal-appearing brain t1 relaxation time predicts disability in early primary progressive multiple sclerosis. Arch. Neurol., 64(3):411–415.
