# Supplementary material for: Quantification of brain proton longitudinal relaxation (T1) in lithium‐treated and lithium‐naïve patients with bipolar disorder in comparison to healthy controls
Source: Bipolar Disord. 2019 Dec 2;23(1):41–8. doi: 10.1111/bdi.12878 (PMC7891392; doi:10.1111/bdi.12878)
Supplement: Supplementary file 3 [file BDI-23-41-s003.docx]

## Supplementary Material C


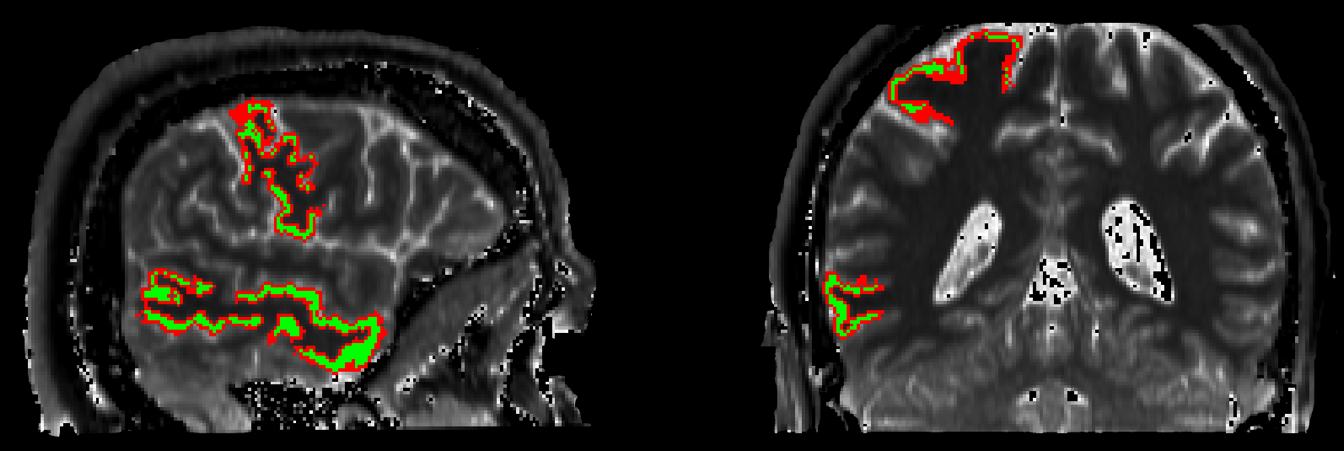


Supplementary Figure C1. Example of ROI erosion on cortical ROIs (Superior temporal gyrus & postcentral gyrus). Red and green regions combined represent original ROI, green region represents ROI following one erosion iteration. Background image: Co-registered T_1_ parameter map.


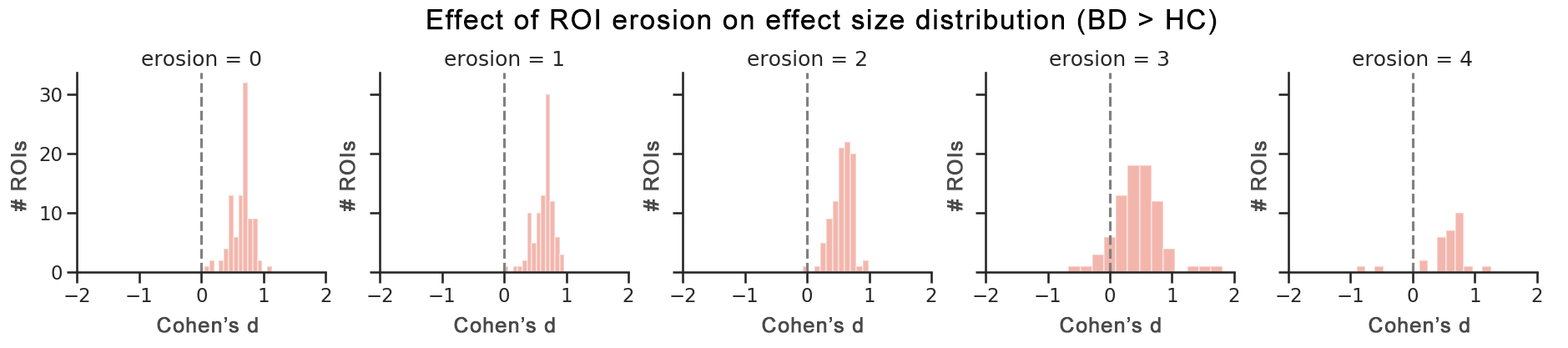


Supplementary Figure C2. Bar plot of Cohen’s d scores for all ROIs following different iterations of erosion (ranging from 0 - 4). Erosion of ROIs does not change overall pattern of results; however, the number of ROIs become reduced at high erosion iterations as small ROIs are lost.

Table C1: Effect size (Cohen’s d), and significance testing for BD>HC comparison for each region of interest (excluding cerebellum) in the Desikan-Killiany atlas.

| Original ROI label | ROI Abbreviation | Cohen's d | Original p value | FDR corrected p value |
| --- | --- | --- | --- | --- |
| Brain-Stem | Brainstem | 0.706318 | 0.016764749 | 0.037236386 |
| CC_Anterior | CC_Ant | 0.613756 | 0.036467494 | 0.055403719 |
| CC_Central | CC_Central | 0.706892 | 0.016680645 | 0.037236386 |
| CC_Mid_Anterior | CC_Mid_Ant | 0.930345 | 0.001980668 | 0.037236386 |
| CC_Mid_Posterior | CC_Mid_Post | 0.684046 | 0.020334195 | 0.04066839 |
| CC_Posterior | CC_Post | 0.749488 | 0.011411593 | 0.037236386 |
| ctx-lh-bankssts | STS | 0.697064 | 0.018172931 | 0.037236386 |
| ctx-lh-caudalanteriorcingulate | CAC | 0.668089 | 0.023295928 | 0.042937593 |
| ctx-lh-caudalmiddlefrontal | CMF | 0.559802 | 0.055592252 | 0.07360101 |
| ctx-lh-cuneus | CN | 0.792458 | 0.007678555 | 0.037236386 |
| ctx-lh-entorhinal | ER | 0.213392 | 0.459032599 | 0.463968433 |
| ctx-lh-frontalpole | FP | 0.377989 | 0.192061908 | 0.203019588 |
| ctx-lh-fusiform | FF | 0.614938 | 0.036122529 | 0.055403719 |
| ctx-lh-inferiorparietal | IP | 0.636541 | 0.030305065 | 0.051072853 |
| ctx-lh-inferiortemporal | IT | 0.53301 | 0.067931477 | 0.088688317 |
| ctx-lh-insula | IS | 0.602967 | 0.03975028 | 0.058383224 |
| ctx-lh-isthmuscingulate | IC | 0.703506 | 0.017181776 | 0.037236386 |
| ctx-lh-lateraloccipital | LO | 0.518764 | 0.075387291 | 0.095762235 |
| ctx-lh-lateralorbitofrontal | LOF | 0.431614 | 0.137269843 | 0.156098142 |
| ctx-lh-lingual | LG | 0.623448 | 0.033723296 | 0.054304145 |
| ctx-lh-medialorbitofrontal | MOF | 0.698018 | 0.018022897 | 0.037236386 |
| ctx-lh-middletemporal | MT | 0.770681 | 0.009401379 | 0.037236386 |
| ctx-lh-paracentral | PCt | 0.699639 | 0.017770687 | 0.037236386 |
| ctx-lh-parahippocampal | PH | 0.348844 | 0.228077368 | 0.23821414 |
| ctx-lh-parsopercularis | PO | 0.607274 | 0.038409986 | 0.057310138 |
| ctx-lh-parsorbitalis | POrb | 0.330499 | 0.253152269 | 0.261497948 |
| ctx-lh-parstriangularis | PT | 0.473662 | 0.10364485 | 0.123324251 |
| ctx-lh-pericalcarine | PCalc | 0.508384 | 0.081242391 | 0.101823797 |
| ctx-lh-postcentral | PoCt | 0.85222 | 0.004333048 | 0.037236386 |
| ctx-lh-posteriorcingulate | PCing | 0.83626 | 0.005060197 | 0.037236386 |
| ctx-lh-precentral | PreCt | 0.740364 | 0.012392156 | 0.037236386 |
| ctx-lh-precuneus | PC | 0.738222 | 0.012633124 | 0.037236386 |
| ctx-lh-rostralanteriorcingulate | RAC | 0.765046 | 0.009901502 | 0.037236386 |
| ctx-lh-rostralmiddlefrontal | RMF | 0.505251 | 0.083082447 | 0.102759869 |
| ctx-lh-superiorfrontal | SF | 0.575566 | 0.049269928 | 0.067121351 |
| ctx-lh-superiorparietal | SP | 0.863171 | 0.003891748 | 0.037236386 |
| ctx-lh-superiortemporal | ST | 0.721004 | 0.014731108 | 0.037236386 |
| ctx-lh-supramarginal | SM | 0.734909 | 0.013014389 | 0.037236386 |
| ctx-lh-temporalpole | TP | -0.00609 | 0.983088407 | 0.983088407 |
| ctx-lh-transversetemporal | TT | 0.815749 | 0.006161376 | 0.037236386 |
| ctx-rh-bankssts | STS | 0.696753 | 0.018222061 | 0.037236386 |
| ctx-rh-caudalanteriorcingulate | CAC | 0.824048 | 0.005691508 | 0.037236386 |
| ctx-rh-caudalmiddlefrontal | CMF | 0.522964 | 0.073120897 | 0.094155676 |
| ctx-rh-cuneus | CN | 0.730383 | 0.013552109 | 0.037236386 |
| ctx-rh-entorhinal | ER | 0.227366 | 0.430312889 | 0.439667517 |
| ctx-rh-frontalpole | FP | 0.434057 | 0.13510332 | 0.156098142 |
| ctx-rh-fusiform | FF | 0.709882 | 0.016249118 | 0.037236386 |
| ctx-rh-inferiorparietal | IP | 0.590477 | 0.043870493 | 0.062482218 |
| ctx-rh-inferiortemporal | IT | 0.430985 | 0.137831338 | 0.156098142 |
| ctx-rh-insula | IS | 0.613499 | 0.036542879 | 0.055403719 |
| ctx-rh-isthmuscingulate | IC | 0.737254 | 0.012743491 | 0.037236386 |
| ctx-rh-lateraloccipital | LO | 0.570307 | 0.051306408 | 0.068897177 |
| ctx-rh-lateralorbitofrontal | LOF | 0.425409 | 0.14289436 | 0.159905594 |
| ctx-rh-lingual | LG | 0.630293 | 0.03189616 | 0.052600686 |
| ctx-rh-medialorbitofrontal | MOF | 0.899645 | 0.002706315 | 0.037236386 |
| ctx-rh-middletemporal | MT | 0.668554 | 0.023204368 | 0.042937593 |
| ctx-rh-paracentral | PCt | 0.681016 | 0.020869136 | 0.040726213 |
| ctx-rh-parahippocampal | PH | 0.433888 | 0.135252964 | 0.156098142 |
| ctx-rh-parsopercularis | PO | 0.679013 | 0.021229622 | 0.040726213 |
| ctx-rh-parsorbitalis | POrb | 0.387229 | 0.181590759 | 0.19620151 |
| ctx-rh-parstriangularis | PT | 0.377852 | 0.192220674 | 0.203019588 |
| ctx-rh-pericalcarine | PCalc | 0.639864 | 0.029487824 | 0.051072853 |
| ctx-rh-postcentral | PoCt | 0.811745 | 0.00640071 | 0.037236386 |
| ctx-rh-posteriorcingulate | PCing | 0.768316 | 0.009608356 | 0.037236386 |
| ctx-rh-precentral | PreCt | 0.708305 | 0.016475449 | 0.037236386 |
| ctx-rh-precuneus | PC | 0.794613 | 0.007524896 | 0.037236386 |
| ctx-rh-rostralanteriorcingulate | RAC | 0.720753 | 0.014763849 | 0.037236386 |
| ctx-rh-rostralmiddlefrontal | RMF | 0.397791 | 0.170161093 | 0.185990032 |
| ctx-rh-superiorfrontal | SF | 0.583706 | 0.046255273 | 0.063941113 |
| ctx-rh-superiorparietal | SP | 0.767342 | 0.009694849 | 0.037236386 |
| ctx-rh-superiortemporal | ST | 0.728537 | 0.013777119 | 0.037236386 |
| ctx-rh-supramarginal | SM | 0.778164 | 0.008772894 | 0.037236386 |
| ctx-rh-temporalpole | TP | 0.412063 | 0.15560727 | 0.172083333 |
| ctx-rh-transversetemporal | TT | 0.843124 | 0.004734555 | 0.037236386 |
| Left-Accumbens-area | Accumbens | 0.649235 | 0.027287104 | 0.048395996 |
| Left-Amygdala | Amygdala | 0.480861 | 0.098625213 | 0.118856026 |
| Left-Caudate | Caudate | 0.622133 | 0.034084517 | 0.054304145 |
| Left-Cerebral-White-Matter | CerebralWM | 0.716641 | 0.015310633 | 0.037236386 |
| Left-Hippocampus | Hippocampus | 0.592008 | 0.043346072 | 0.062482218 |
| Left-Pallidum | Pallidum | 0.636055 | 0.03042638 | 0.051072853 |
| Left-Putamen | Putamen | 0.502397 | 0.084789096 | 0.103508766 |
| Left-Thalamus-Proper | Thalamus | 0.654859 | 0.026037222 | 0.047067286 |
| Right-Accumbens-area | Accumbens | 0.723108 | 0.014458827 | 0.037236386 |
| Right-Amygdala | Amygdala | 0.7345 | 0.013062166 | 0.037236386 |
| Right-Caudate | Caudate | 0.776072 | 0.008944569 | 0.037236386 |
| Right-Cerebral-White-Matter | CerebralWM | 0.748534 | 0.01151071 | 0.037236386 |
| Right-Hippocampus | Hippocampus | 0.704681 | 0.01700639 | 0.037236386 |
| Right-Pallidum | Pallidum | 0.721288 | 0.014694089 | 0.037236386 |
| Right-Putamen | Putamen | 0.585452 | 0.045629735 | 0.063941113 |
| Right-Thalamus-Proper | Thalamus | 0.739368 | 0.012503697 | 0.037236386 |
